# Chlorophyllin Bait Formulation and Exposure to Different Spectrum of Visible Light on the Reproduction of Infected/Uninfected Snail* Lymnaea acuminata*

**DOI:** 10.1155/2016/9795178

**Published:** 2016-01-27

**Authors:** Navneet Kumar, D. K. Singh, Vinay Kumar Singh

**Affiliations:** Malacology Laboratory, Department of Zoology, D.D.U. Gorakhpur University, Gorakhpur, Uttar Pradesh 273009, India

## Abstract

Fasciolosis is a waterborne disease, caused by* Fasciola* species. Snail* Lymnaea acuminata* is an intermediate host of these flukes. Control of snail population is major tool in reducing the incidences. Variation in light intensity and wavelength caused significant changes in reproduction pattern of snails. Maximum fecundity was noted with bait containing carbohydrate (starch, 468 ± 0.10/20 snails) or amino acid (serine, 319 ± 0.29/20 snails) as attractant. Sublethal feeding of chlorophyllin bait with starch or serine attractant to infected and uninfected snails caused significant reduction in fecundity, hatchability, and survivability. These significant changes are observed in snails exposed to different spectral band of visible light and sunlight. Maximum fecundity of 536 ± 2.0 and minimum of 89.3 ± 0.4 were noted in snails not fed with bait and exposed to sunlight and red spectral band, respectively. There was complete arrest in the fecundity of infected and uninfected snails and no survivability of uninfected snails after 48 h feeding with bait containing chlorophyllin + attractant. Minimum hatchability (9.25 ± 0.5) was noted in red light exposed, chlorophyllin + starch fed infected snails and hatching period of bait fed snails was prolonged. Conclusively, chlorophyllin bait and red light reduce reproduction capacity in snails.

## 1. Introduction

Fresh water snail* Lymnaea acuminata* is the intermediate host of* Fasciola hepatica* and* F. gigantica* [1, 2], which caused endemic fasciolosis in cattle population of eastern Uttar Pradesh [3]. Use of aquatic plants as fodder is the primary cause of fluke infection. About 94% of buffaloes slaughtered in the local slaughter houses are infected with heavy infection of liver flukes [4, 5]. Transmission of fasciolosis can be controlled by reducing the population of vector snails by the use of molluscicide. Now it has been advocated that use of synthetic molluscicide is not environmentally safe [3, 6]. Alternatively, plant based molluscicides are used against vector snails. Behavioral response of snails against light has attracted the attention of biologist working in the field of chronobiology [7]. Light intensity and wavelength are the important factors, which affect the physiology of organism [8], behavior [9, 10], and reproduction of organism [11]. Recently, Tripathi et al. [12] had noted that snail* L. acuminata* is more attracted towards red spectral band of visible light. Chlorophyllin, a derivative of chlorophyll, is potent larvicides against insect and helminth larvae in sunlight [13–16]. Snail* L. acuminata* breeds round the year. Snail reproduces 169–172 eggs a day; likewise an adult liver fluke* Fasciola gigantica* reproduces 10–20 thousand eggs a day [17], and both the parasite and the host reproduce tremendously to maintain their existence. In the present study chlorophyllin baits will be fed to infected and uninfected snail* L. acuminata* and incubated for 3 hours. Thereafter, the fecundity of infected and uninfected snails will be counted to elucidate the effect of chlorophyllin on the reproduction pattern of chlorophyllin fed snails.

## 2. Materials and Methods

### 2.1. Collection of Snails

The adult snails (2.25 ± 0.25 cm in length) were collected from Maheshra Lake Gorakhpur, India. The field collected adult snail* L. acuminata* were acclimatized for 72 h, in dechlorinated tap water, at 22°C to 24°C. The pH of water was 7.2 to 7.3 and dissolved oxygen, free carbon dioxide, and bicarbonate alkalinity were 6.2 to 7.2 mg/L, 5.2–6.3 mg/L,  and 102–106 mg/L, respectively. Acclimated snails were used in the experiment. Infected and uninfected snails were identified by the method of Sunita et al. [18].

### 2.2. Test Material

Chlorophyllin was prepared by the method of Wohllebe et al. [13]. Chlorophyll was extracted from deep-frozen spinach leaves by adding 100% ethanol to the extract and incubating it (for about 2 h) at 55°C; then CaCO_3_ was added to avoid transformation of chlorophyllin into pheophytin (about 1 g/1 kg plant material). The extract was filtered and petroleum benzene was added. The upper lipophilic phase was taken and saponified with methanolic KOH, which converted the chlorophyll into water soluble chlorophyllin. Attractants (carbohydrate and amino acid) were purchased from Sigma Chemical Co., USA.

### 2.3. Preparation of Bait

The bait pellets were prepared by the method of Madsen [19] as modified by Tiwari and Singh [20]. Carbohydrate starch (10 mM) and serine (20 mM) were added to 100 mL of water in 2% agar solution, separately. Chlorophyllin was added inside the bait. The mixture was stirred constantly for 30 min and spread at a uniform thickness (5 mm); after cooling the baits containing chlorophyllin were cut out measuring 5 mm in diameter. These baits of chlorophyllin + starch/serine were fed to the snails; thereafter the reproductive behavior of snails was noted.

### 2.4. Protocol of Light Source

Xenon arc lamp (500 w) was used as visible light source. Spectral responses from 400 to 650 nm were produced with the help of interference colour filters. Light intensity was measured against each filter and then output of light was adjusted to get the equal irradiance of 500 Wm^−2^ at each band to study their effect on snail reproduction [12].

### 2.5. Fecundity Experiment

Group of 20 snails kept in glass jars containing 3 liters of dechlorinated tap water were fed separately with bait containing sublethal concentration of chlorophyllin and exposed to visible light. Total number of eggs masses and eggs laid by the each group of snails were counted every 24 h up to 96 h.* L. acuminata* laid eggs in the form of elongated gelatinous capsules (egg mass or egg strings) on the lower surface of leaves of aquatic vegetation. The egg laid by the snail* L. acuminata* was collected in glass petri dish. The hatching of the eggs was studied under the microscope, in light and dark condition. These egg masses may have 2-3 rows of eggs, the number of eggs ranging from 5 to 200 or sometimes even more. Capsules containing egg forms treated and control groups were separated carefully from the lower surface of lotus leaves and incubated at 30°C in petri dish. The development of embryo was observed under the microscope up to their hatching period. Hatchability was noted in the eggs laid after 24 h to 96 h feeding of bait. Dead embryos (lack embryonic movements and become opaque) were removed immediately to avoid any contamination. After hatching the miniature snails were reared on lotus leaves and their survival was observed.

Four experiments were done simultaneously to notice the effect of chlorophyllin on the reproduction pattern of snail* L. acuminata*. In experiment number one (control a) snails were exposed to different spectral band of visible light/sunlight/no light (dark) for 8 h without feeding. Thereafter, fecundity, hatchability, and survivability of miniature snails were noted. In experiment number two (control b) snails were fed with the bait containing different carbohydrate or amino acids in laboratory condition and, thereafter, fecundity, hatchability, and survival of miniature snails were noted. In experiment number three infected snails were fed with bait containing chlorophyllin + starch/serine and exposed to red light or sunlight. In experiment number four uninfected snails were fed with chlorophyllin bait; thereafter, fecundity, hatchability, and survivability of miniature snails were noted.

### 2.6. Statistical Analysis

A Student *t*-test was applied to determine the significant (*P* < 0.005) differences between treated and control animals. Product moment correlation coefficient was applied between exposure time and fecundity and survival of hatched snails [21].

## 3. Result

The present study clearly indicates that there was a significant reduction in reproduction of snails fed chlorophyllin bait and exposed to red light after 3 h. Amongst the entire spectral band, red light was more effective in reducing the fecundity of snails. Although fecundity of snails significantly decreases in all the spectrum of light, maximum reduction was noted in red light (24 h 89.3 ± 0.4/20 snails and 96 h fecundity 0), hatchability, and survival of snail. The hatching period of eggs laid by snail in control experiment (a) was prolonged in red spectral band (14–17 days) with respect to control (no light 10–12 days) (Table 1). Fecundity was enhanced, when snails were fed with bait containing different carbohydrate/amino acids in laboratory condition. Starch (468 ± 0.1 and 96 h 84 ± 0.26) and serine (319 ± 0.29 and 96 h 70 ± 0.72) bait fed snails had the maximum fecundity amongst different carbohydrate/amino acids compared to control (24 h 200 ± 0.23 and 96 h 98 ± 0.31), respectively. Survival of miniature snails whose parents were fed with serine bait (24 h 167 ± 0.42 and 96 h survival 127 ± 0.97) was maximum in comparison to control (24 h 100 ± 1.2 and 96 h survival 85 ± 1.2) (Table 2).

Sublethal feeding of bait containing 24 h LC_50_ of 40% and 80% chlorophyllin + starch/serine significantly reduced the reproductive capacity (fecundity, hatchability, and survivability) of* L. acuminata* snails. Feeding of chlorophyllin baits caused dose dependent change in fecundity, hatchability, and survivability of snail's exposure to 660 nm wavelength. Reproductive ability of snails was reduced to the maximum when they were fed bait containing chlorophyllin and exposed to natural sunlight/red light (660 nm). Feeding of bait formulation containing 24 h LC_50_ of 40% chlorophyllin and serine in red light causes maximum reduction in reproduction of infected snails (24 h 99.05 ± 0.4 and 48 h survival 11 ± 0.28). Hatching period was prolonged to 15–17 days in red light and 15–19 days in natural sunlight with respect to 7–9 days in control. No survivability of newly hatched snails was noted in treated snails after 48 h; the newly hatched snails in treated group were slow and had smaller tentacles as compared with those of control group. Feeding of bait containing 24 h LC_50_ of 80% chlorophyllin arrested hatchability and survivability (Table 3). Sublethal feeding of bait containing 24 h LC_50_ of 40% and 80% of chlorophyllin + serine to uninfected snails resulted in minimum fecundity in sunlight + serine (24 h 74.25 ± 0.01 and 48 h fecundity 10.65 ± 0.94) and red light + starch (24 h 56.25 ± 0.84 and 48 h fecundity 4.25 ± 0.25) as compared to control (24 h 206 ± 0.88 and 96 h fecundity 140 ± 2.0). 24 h LC_50_ of 80% chlorophyllin feeding of bait heavily reduces the fecundity of uninfected snail (after 48 h). The control snail laid equal sized eggs in two rows in gelatinous strings (Table 4).

## 4. Discussion

Caudodorsal cells (CDCs) in the Lymnaeidae snails are the main centre to control the egg laying [22, 23]. Seasonal variation in egg laying pattern of* Lymnaea* is noted by various works [24, 25]. Ter Maat et al. [26] reported the role of light perceived through ocular or nonocular photoreception on induced egg laying pattern in snail* Lymnaea stagnalis*. According to them nonocular photoreception can modulate the egg laying pattern [27]. This light photoreception reaches the caudodorsal cells through lipochondria present in the intracellular neuronal organelles, which contain carotenoids [28, 29]. Effects of different abiotic factors, namely, temperature, pH, dissolved O_2_, and free CO_2_, on egg laying pattern of snails are reported by different workers [23, 25]. There is no study on the effect of different spectral bands of visible light on the egg laying pattern of Lymnaeidae snails.

Present study clearly demonstrates a significant reduction in the fecundity of* L. acuminata* snails exposed to different spectral band of visible light after feeding of bait containing chlorophyllin + amino acid. Although red light attracts maximum number of snails [12, 30], it reduces the reproductive capacity of both infected and uninfected snails. It is noted that aquatic insects are more sensitive to red light than other invertebrates. Red light penetrates water column more than another band of visible light [31]. According to Bruce and Shardlow [32] arthropods, Mollusca, and some worms have eyes that are extremely sensitive to different spectral band of light.

The reduction in the hatchability of* L. acuminata* snails exposed to the different spectra band of visible light may be due to more interference of reactive oxygen, a product of chlorophyllin, with the embryonic growth and development of the snails [33]. Young hatched snail shows delay in attaining maturity in both treated and control groups exposed to different spectral band of visible light. Sunlight having different spectral bands affects the behavior, biological rhythms, and physiological functions of snails [34, 35]. Reproduction of mosquito is prevented through blue light irradiation on eggs, larva, and pupa [7]. Blue (470 nm) and red (660 nm) spectral band may stimulate the production of reactive oxygen substrate [36]. Yin et al. [37] suggested that many microbial cells are sensitive to blue light as a result of the accumulation of photosensitizers such as porphyrins and flavins.* Fasciola* infected snail requires higher feeding due to larval growth of parasite in their body and higher accumulation of chlorophyllin. It produces more toxic reactive oxygen than uninfected snails. Consequently, negative effect of chlorophyllin bait feeding was noted on fecundity, hatchability, and survival of snails.

## 5. Conclusion

It can be concluded from the present study that the snails have a capacity to monitor photo- and chemostimulus. At higher intensity, snails attracted by red light caused the alteration of reproductive capacity with bait containing chlorophyllin. These behavioral responses of snail against both stimuli will be a new tool in managing the population of snails below threshold level to minimize fasciolosis.

## Figures and Tables

**Table 1:** Effect on fecundity, hatchability, and survivability of snails kept for 8 h exposure in different visible spectrum of light.

Exposure	Fecundity %				Hatchability %	Survivability %		
	24 h	48 h	72 h	96 h		24 h	48 h	72 h
Control (no light)	250 ± 0.2^+*∗*^	197 ± 0.9^+*∗*^	102 ± 0.7^+*∗*^	50 ± 0.40^+*∗*^	34 ± 0.63^+*∗*^ (10–12)	45 ± 1.8^+*∗*^	25 ± 1.6^+*∗*^	12 ± 0.96^+*∗*^
Red light	89.3 ± 0.4^+*∗*^	63.2 ± 0.2^+*∗*^	20.3 ± 0.8^+*∗*^	0	32.6 ± 0.91^+^ (14–17)	22 ± 0.9^+*∗*^	13 ± 0.01^+*∗*^	0
Sunlight	536 ± 2.0^+*∗*^	316 ± 0.14^+*∗*^	398 ± 0.04^+*∗*^	300 ± 1.6^+*∗*^	396 ± 1.92^+*∗*^ (9–13)	245 ± 1.8^+*∗*^	192 ± 2.6^+*∗*^	157 ± 0.97^+*∗*^
Blue light	185 ± 1.8^+*∗*^	85 ± 0.04^+*∗*^	73 ± 3.8^+*∗*^	42 ± 1.9^+*∗*^	96 ± 1.92^+*∗*^ (10–14)	55 ± 1.8^+*∗*^	40 ± 1.8^+*∗*^	0
Green light	251 ± 0.03^+*∗*^	188 ± 0.24^+*∗*^	142 ± 3.7^+*∗*^	66 ± 0.2^+*∗*^	82 ± 0.25^+*∗*^ (9–14)	76.6 ± 0.2^+*∗*^	60.5 ± 0. 9^+*∗*^	55 ± 1.81^+*∗*^
Yellow light	291 ± 0.03^+*∗*^	146 ± 0.28^+*∗*^	93 ± 0.2^+*∗*^	56 ± 3.2^+*∗*^	44 ± 0.47^+*∗*^ (10–12)	21.6 ± 0.9^+*∗*^	38 ± 1.0^+*∗*^	35 ± 1.1^+*∗*^

Each experiment was repeated six times and the values of fecundity, hatchability, and survival were the mean of six replicates. ^*∗*^Significant (*P* < 0.05) difference was noted when Student's *t*-test was applied to treated and control groups.

^+^Product moment correlation coefficient showed that there was significant (*P* < 0.05) negative correlation in between exposure period and fecundity of *L. acuminata* snail. (0) shows no fecundity, hatchability, and survival.

**Table 2:** Fecundity, hatchability, and survivability of snail (*L. acuminata)* 2 h exposure to attractants carbohydrate (10 mM) and amino acid (20 mM) in bait.

Exposure	Fecundity %				Hatchability %	Survival %			
	24 h	48 h	72 h	96 h		24 h	48 h	72 h	96 h
Control	200 ± 0.23^+*∗*^	80 ± 0.25^+*∗*^	155 ± 1.2^+*∗*^	98 ± 0.31^*∗*^	150 ± 0.25^+*∗*^ (7–9)	100 ± 1.2^+*∗*^	99 ± 0.21^+*∗*^	89 ± 0.12^+*∗*^	85 ± 1.2^+*∗*^
Maltose	283 ± 0.25^+*∗*^	218 ± 0.33^+*∗*^	123 ± 0.13^+*∗*^	14 ± 0.68^*∗*^	189 ± 0.36^+*∗*^ (8–10)	91 ± 1.04^+*∗*^	143 ± 0.39^+*∗*^	125 ± 0.11^+*∗*^	13 ± 0.26^+*∗*^
Glucose	276 ± 0.93^+*∗*^	218 ± 0.27^+*∗*^	123 ± 0.08^+*∗*^	14 ± 0.68^*∗*^	183 ± 0.25^+*∗*^ (10–13)	151 ± 0.41^+*∗*^	113 ± 0.22^+*∗*^	107 ± 0.91^+*∗*^	95 ± 1.8^+*∗*^
Sucrose	258 ± 0.76^+*∗*^	224 ± 1.1^+*∗*^	108 ± 5.2^+*∗*^	79 ± 0.63^*∗*^	151 ± 0.25^+*∗*^ (8–13)	140 ± 1.81^+*∗*^	125 ± 1.81^+*∗*^	115 ± 1.8^+*∗*^	91.3 ± 0.24^+*∗*^
Starch	468 ± 0.1^+*∗*^	290 ± 0.2^+*∗*^	143 ± 19^+*∗*^	84 ± 0.26^*∗*^	162 ± 0.35^+*∗*^ (11–14)	142 ± 0.20^+*∗*^	121 ± 0.14^+*∗*^	112 ± 0.91^+*∗*^	95 ± 1.8^+*∗*^
Serine	319 ± 0.29^+*∗*^	232 ± 0.21^+*∗*^	81 ± 0.20^+*∗*^	70 ± 0.72^*∗*^	239 ± 0.34^+*∗*^ (8–12)	167 ± 0.42^+*∗*^	154 ± 1.91^+*∗∗*^	143 ± 0.26^+*∗*^	127 ± 0.97^+*∗*^
Aniline	254 ± 0.70^+*∗*^	181 ± 0.81^+*∗*^	110 ± 1.8^+*∗*^	51 ± 0.40^*∗*^	197 ± 0.27^+*∗*^ (7–10)	186 ± 1.99^+*∗*^	145 ± 1.81^+*∗*^	127 ± 0.91^+*∗*^	115 ± 0.18^+*∗*^
Proline	71 ± 0.68^+*∗*^	201 ± 0.27^+*∗*^	125 ± 0.78^+*∗*^	109 ± 4.3^*∗*^	203 ± 1.37^+*∗*^ (10–15)	164 ± 0.24^+*∗*^	118 ± 0.29^+*∗*^	107 ± 0.91^+*∗*^	85 ± 1.8^+*∗*^

Each experiment was repeated six times and the values of fecundity, hatchability, and survival were the mean of six replicates. Values in parenthesis indicate the days. ^*∗*^Significant (*P* < 0.05) difference was noted when Student's *t*-test was applied to treated and control groups.

^+^Product moment correlation coefficient showed that there was significant (*P* < 0.05) negative correlation in between exposure period and fecundity of *L. acuminata* snail.

**Table 3:** Effect of sublethal doses (40% and 80% of 24 h LC_50_) of chlorophyllin bait containing serine and starch (in red light/sunlight) on the fecundity, hatchability, and survival of the infected snails* L. acuminata* eggs.

Exposure	LC_50_ 24 h	Sublethal dose (mg/L)	Fecundity %				Hatchability %	Survivability %		
Uninfected snails			24 h	48 h	72 h	96 h		24 h	48 h	72 h
Control	—	—	220 ± 0.15^+*∗*^	180 ± 1.2^+*∗*^	150 ± 1.3	100 ± 2.0	95.2 ± 2.0^+*∗*^ (7–9)	96 ± 0.3^+*∗*^	89 ± 3.2^+*∗*^	50 ± 0.3

Red light starch	7.05	40% (2.82)	122.5 ± 0.84^+*∗*^	67.5 ± 0.48^+*∗*^	0	0	9.25 ± 0.5^+*∗*^ (9–11)	88 ± 0.5^+*∗*^	36.4 ± 0.2^+*∗*^	0
		80% (5.64)	106 ± 0.81^+*∗*^	37.5 ± 0.48^+*∗*^	0	0	0	0	0	0

Red light serine	7.89	40% (3.15)	99.5 ± 0.04^+*∗*^	11.2 ± 0.28^+*∗*^	0	0	19.25 ± 0.8^+*∗*^ (15–17)	37.4 ± 0.6^+*∗*^	47.1 ± 0.5^+*∗*^	0
		80% (6.31)	80.25 ± 0.16^+*∗*^	18.5 ± 0.10^+*∗*^	0	0	0	0	0	0

Sunlight starch	9.89	40% (3.94)	135 ± 0.96^+*∗*^	32.25 ± 0.07^+*∗*^	0	0	23.5 ± 0.92^+*∗*^ (10–12)	42.6 ± 0.20^+*∗*^	37.4 ± 0.5^+*∗*^	0
		80% (7.88)	108 ± 0.28^+*∗*^	29.5 ± 0.19^+*∗*^	0	0	0	0	0	0

Sunlight serine	10.93	40% (4.37)	67 ± 0.93^+*∗*^	32.5 ± 0.45^+*∗*^	0	0	28.5 ± 0.09^+*∗*^ (12-13)	42.6 ± 0.2^+*∗*^	36.4 ± 0.5^+*∗*^	0
		80% (8.74)	55.25 ± 1.0^+*∗*^	14.25 ± 0.8^+*∗*^	0	0	21.5 ± 0.10^+*∗*^ (15–19)	43 ± 0.4^+*∗*^	31 ± 0.1^+*∗*^	0

Each experiment was repeated six times and the values of fecundity, hatchability, and survival were the mean of six replicates. ^*∗*^Significant (*P* < 0.05) difference was noted when Student's *t*-test was applied to treated and control groups.

^+^Product moment correlation coefficient showed that there was significant (*P* < 0.05) negative correlation in between exposure period and fecundity of *L. acuminata* snail. (—) shows no dose; (0) shows no fecundity, hatchability, and survivability.

**Table 4:** Effect of sublethal (40% and 80% of 24 h LC_50_) chlorophyllin in bait containing serine and starch (in red light/sunlight) on the fecundity, hatchability, and survival of the uninfected snails *L. acuminata* eggs.

Exposure	LC_50_ 24 h	Sublethal dose (mg/L)	Fecundity				Hatchability %	Survivability %		
Infected snails			24 h	48 h	72 h	96 h		24 h	48 h	72 h
Control	—	—	206 ± 0.88^*∗*^	185 ± 0.92^+*∗*^	150 ± 0.2	140 ± 2.0	96.25 ± 0.21^+*∗*^ (7–9)	85 ± 0.30^+*∗*^	79 ± 0.10^+*∗*^	69 ± 0.12^+*∗*^

Red light starch	9.34	40% (3.73)	114 ± 0.74^*∗*^	67.5 ± 0.48^+*∗*^	0	0	10.75 ± 0.06^+*∗*^ (15–19)	30 ± 0.20^+*∗*^	25 ± 0.11^+*∗*^	15 ± 0.1^+*∗*^
		80% (7.47)	56.25 ± 0.84^*∗*^	4.25 ± 0.92^+*∗*^	0	0	0	0	**0**	**0**

Red light serine	10.93	40% (4.37)	95.5 ± 0.99^*∗*^	29.25 ± 0.19^+*∗*^	0	0	16.25 ± 0.67^+*∗*^ (14–18)	33 ± 0.11^+*∗*^	26 ± 0.20^+*∗*^	12 ± 0.01^+*∗*^
		80% (8.74)	64.75 ± 0.58^*∗*^	20 ± 0.42^+*∗*^	0	0	0	0	0	0

Sunlight starch	11.05	40% (4.42)	74.25 ± 0.01^*∗*^	10.65 ± 0.94^+*∗*^	0	0	21.25 ± 0.28^+*∗*^ (12–15)	40 ± 0.12^+*∗*^	32 ± 0.21^+*∗*^	12 ± 0.1^+*∗*^
		80% (8.82)	60.5 ± 0.51^*∗*^	18.75 ± 0.84^+*∗*^	0	0	21.25 ± 0.51^+*∗*^ (15–17)	32 ± 0.12^+*∗*^	22 ± 0.01^+*∗*^	12 ± 0.2^+*∗*^

Sunlight serine	11.57	40% (4.28)	99.25 ± 0.47^*∗*^	22.5 ± 0.13^+*∗*^	0	0	27.5 ± 0.48^+*∗*^ (12–15)	46 ± 1.0^+*∗*^	34 ± 0.20^+*∗*^	16 ± 0.2^+*∗*^

Each value is mean ± S.E of six replicates. Each replicate represents the eggs laid by the group of 20 snails. ^*∗*^Significant (*P* < 0.05) difference was noted when Student's *t*-test was applied to treated and control groups.

^+^Product moment correlation coefficient showed that there was significant (*P* < 0.05) negative correlation in between exposure period and fecundity of snail *L. acuminata.* (—) shows no dose; (0) shows no fecundity, hatchability, and survivability.

## References

[1] Mas-Coma S., Valero M. A., Bargues M. D. (2009). Fasciola, lymnaeids and human fascioliasis, with a global overview on disease transmission, epidemiology, evolutionary genetics, molecular epidemiology and control. *Advances in Parasitology*.

[2] Imani-Baran A., Yakhchali M., Viayeh R. M., Paktarmani R. (2012). Molecular study for detecting the prevalence of *Fasciola gigantica* in field-collected snails of *Radix gedrosiana* (Pulmonata: Lymnaeidae) in northwestern Iran. *Veterinary Parasitology*.

[3] Agarwal R. A., Singh D. K. (1988). Harmful gastropods and their control. *Acta Hydrochimica et Hydrobiologica*.

[4] Singh O., Agarwal R. A. (1981). Toxicity of certain pesticides to two economic species of snails in Northern India. *Journal of Economic Entomology*.

[5] Singh D. K., Singh V. K., Kumar P. (2012). *Pestiferous Gastropods and Their Control*.

[6] Singh A., Singh D. K., Misra T. N., Agarwal R. A. (1996). Molluscicides of plant origin. *Biological Agriculture and Horticulture*.

[7] Hori M., Shibuya K., Sato M., Saito Y. (2014). Lethal effects of short-wavelength visible light on insects. *Scientific Reports*.

[8] Haim A., Shanas U., Zubidad A. E. S., Scantelbury M. (2005). Seasonality and seasons out of time—the thermoregulatory effects of light interference. *Chronobiology International*.

[9] Frank K. D., Rich C., Longcore T. (2006). Effects of artificial night lighting on moths. *Ecological Consequences of Artificial Night Lighting*.

[10] Eisenbeis G., Rich C., Longcore T. (2006). Artificial night lighting and insects: attraction of insects to streetlamps in a rural setting in Germany. *Ecological Consequences of Artificial Night Lighting*.

[11] Witherington B. E. (1992). Behavioral responses of nesting sea turtles to artificial lighting. *Herpetologica*.

[12] Tripathi A. P., Singh V. K., Singh D. K. (2013). Behavioral responses of the snail *Lymnaea acuminata* towards photo and chemo attractants: a new step in control program of fasciolosis. *International Journal of Zoology*.

[13] Wohllebe S., Richter R., Richter P., Häder D.-P. (2009). Photodynamic control of human pathogenic parasites in aquatic ecosystems using chlorophyllin and pheophorbid as photodynamic substances. *Parasitology Research*.

[14] Wohllebe S., Richter P., Häder D.-P. (2012). Chlorophyllin for the control of *Ichthyophthirius multifiliis* (Fouquet). *Parasitology Research*.

[15] Erzinger G. S., Wohllebe S., Vollrath F. (2011). Optimizing conditions for the use of chlorophyll derivatives for photodynamic control of parasites in aquatic ecosystems. *Parasitology Research*.

[16] Singh D. J., Singh D. K. (2015). Toxicity of chlorophyllin in different wavelengths of visible light against *Fasciola gigantica* larvae. *Journal of Photochemistry and Photobiology B: Biology*.

[17] Dalton J. P. (1999). *Fasciolosis*.

[18] Sunita K., Kumar P., Singh V. K., Singh D. K. (2013). Larvicidal activity of azadirachtin against * Fasciola* larva. *The Ecoscan*.

[19] Madsen H. A. (1992). A comparative study on the food-locating ability of *Helisoma duryi, Biomphalaria camerunensis* and *Bulinus truncatus* (Pulmonata: Planorbidae). *Journal of Applied Ecology*.

[20] Tiwari F., Singh D. K. (2004). Behavioural responses of the snail *Lymnaea acuminata* to carbohydrates in snail-attractant pellets. *Naturwissenschaften*.

[21] Sokal R. R., Rohlf F. J. (1973). *Introduction to Biostatistics*.

[22] Ter Maat A., Pieneman A. W., Goldschmeding J. T., Smelik W. F. E., Ferguson G. P. (1989). Spontaneous and induced egg laying behavior of the pond snail, *Lymnaea stagnalis*. *Journal of Comparative Physiology A*.

[23] Srivastava A. K., Singh D. K., Singh V. K. (2014). Influence of abiotic factor on anti-reproductive activity of bait containing papain in *Lymnaea acuminata*. *Annual Research & Review in Biology*.

[24] Wayne N. L. (2001). Regulation of seasonal reproduction in mollusks. *Journal of Biological Rhythms*.

[25] Jigyasu H. V., Singh V. K. (2010). Effect of environmental factors on the fecundity, hatchability and survival of snail *Lymnaea* (Radix) *acuminata* (Lamarck): vector of fascioliasis. *Journal of Water and Health*.

[26] Ter Maat A., Pieneman A. W., Koene J. M. (2012). The effect of light on induced egg laying in the simultaneous hermaphrodite *Lymnaea stagnalis*. *Journal of Molluscan Studies*.

[27] Lyons L. C., Rawashdeh O., Eskin A. (2006). Non-ocular circadian oscillators and photoreceptors modulate long term memory formation in Aplysia. *Journal of Biological Rhythms*.

[28] Baur P. S., Brown A. M., Rogers T. D., Brower M. E. (1977). Lipochondria and the light response of *Aplysia* giant neurons. *Journal of Neurobiology*.

[29] Gotow T., Nishi T. (2009). A new photosensory function for simple photoreceptors, the intrinsically photoresponsive neurons of the sea slug *Onchidium*. *Frontiers in Cellular Neuroscience*.

[30] Kumar N., Singh V. K. (2015). Bait formulations of chlorophyllin against infected/uninfected *Lymnaea acuminata* in red and sunlight. *International Journal of Zoological Research*.

[31] Heise B. A. (1992). Sensitivity of mayfly nymphs to red light: implications for behavioural ecology. *Freshwater Biology*.

[32] Bruce C. B., Shardlow M. (2011). *A Review of the Impact of Artificial Light on Invertebrates*.

[33] Singh K., Singh V. K. (2015). Anti-reproductive activity of chlorophyllin on fresh water snail *Lymnaea acuminata*. *Research Journal of Parasitology*.

[34] Shuboni D., Yan L. (2010). Nighttime dim light exposure alters the responses of the circadian system. *Neuroscience*.

[35] Hölker F., Wolter C., Perkin E. K., Tockner K. (2010). Light pollution as a biodiversity threat. *Trends in Ecology and Evolution*.

[36] DeRosa M. C., Crutchley R. J. (2002). Photosensitized singlet oxygen and its applications. *Coordination Chemistry Reviews*.

[37] Yin R., Dai T., Avci P. (2013). Light based anti-infectives: ultraviolet C irradiation, photodynamic therapy, blue light, and beyond. *Current Opinion in Pharmacology*.

